# Electrospun poly(ε-caprolactone)/silver nanoparticle nanofibrous scaffolds with antibacterial activity for wound-dressing applications

**DOI:** 10.1039/d5ra06746d

**Published:** 2025-10-10

**Authors:** Ali L. Alfalluji, Qasim Shakir Kadhim, Ausama AbedAlkhadum Mahdi

**Affiliations:** a College of Basic Education, University of Babylon Babylon 51002 Iraq ali.alfalluji@uobabylon.edu.iq

## Abstract

Silver nanoparticle (AgNP)-based nanofibers are widely used in biomedical applications for their broad-spectrum activity and biocompatibility. This study aims to biofabricate and characterize electrospun polycaprolactone (PCL) nanofibers loaded with biosynthesized AgNPs, with the goal of evaluating their structural properties and antibacterial effectiveness for potential use in antibacterial wound-dressing applications. The biosynthesis of AgNPs was achieved using an aqueous extract of Piper nigrum leaves. Nanocomposite membranes at different AgNP concentrations (0.04, 0.4, and 1 wt%) were prepared to investigate their physicochemical and antibacterial properties. Morphological characterization confirmed bead-free, continuous fiber formation, with reduced fiber diameters upon increasing AgNP content. AFM results revealed enhanced surface roughness. FTIR spectra indicated improved hydrophilicity and successful chemical incorporation of AgNPs. Mechanical testing demonstrated increased tensile strength at 0.4 wt% AgNPs, followed by a decline due to nanoparticle agglomeration at 1 wt%. Contact angle measurements confirmed a significant shift toward hydrophilicity with higher AgNP concentrations. Antibacterial assays revealed strong inhibition against *Escherichia coli* and *Staphylococcus aureus*, with the 1 wt% AgNP scaffold producing the most prominent zones of inhibition. These results suggest that PCL-AgNP nanofibers are a promising antibacterial platform for biomedical applications, particularly as infection–preventive wound-dressing materials.

## Abstract 2

Antibacterial electrospun nanofibrous scaffolds of poly(ε-caprolactone) embedded with silver nanoparticles for advanced wound dressing applications.

## Graphical abstract

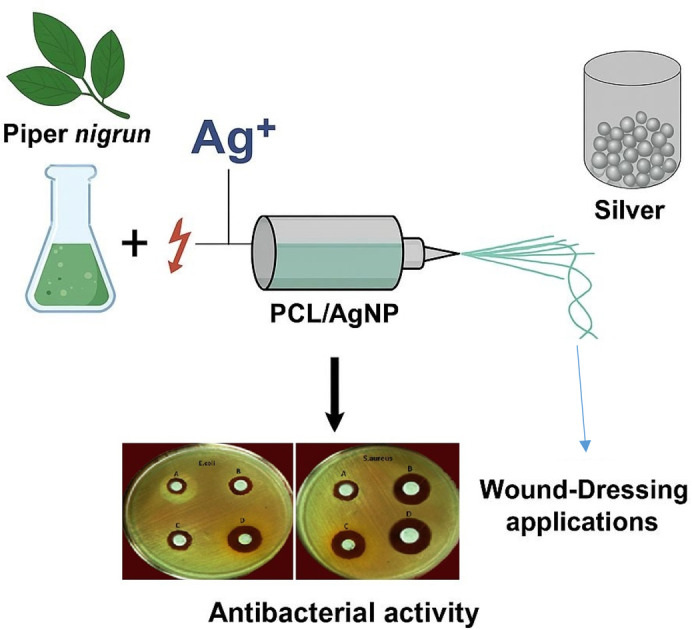

## 1. Introduction

Infection control remains a critical challenge in wound management and biomedical device implantation. Open wounds are highly vulnerable to bacterial colonization, which can ruin healing and trigger severe complications.^1^ Traditional dressings, such as cotton gauze, function mainly as passive barriers and typically lack active antimicrobial properties or the capacity to surrogate a good enough antibacterial.^2,3^ The prevalence of antibiotic-resistant bacteria further intensifies this concern and necessitates urgent and innovative infection–preventive solutions. Accordingly, biomaterial research increasingly aims to develop active antimicrobial scaffolds that both protect wounds and actively inhibit microbial growth. One effective strategy involves integrating antimicrobial nanoparticles into polymer-based nanofibers, transforming wound dressings into physical barriers that also support targeted antimicrobial delivery.^4^ Thereafter, the approach supports the development of scaffolds that simultaneously prevent infection and promote healing, which in a broader sense, push regenerative medicine beyond the constraints of traditional wound care and reduce reliance on systemic antibiotics, that, in its full picture, disturb the wound healing process.

Electrospinning is a common approach used to fabricate non-woven nanofibrous meshes, formed by drawing a polymer solution into ultrafine fibers using a high-voltage electric field to create a Taylor cone and resulting jet. The resulting mats show high porosity and large surface-to-volume ratios, closely mimicking native extracellular matrix (ECM) structures and promoting cell adhesion, oxygen exchange, and nutrient transfer, and other ECM-related features that are highly useful for wound-dressing applications.^5–9^ Importantly, such porous architectures facilitate effective absorption of wound exudate and serve as microbial barriers, therefore limiting pathogen penetration while permitting moisture vapor permeability. Electrospinning also allows the incorporation of bioactive compounds, including drugs, growth factors, or nanoparticles, either within the fiber core or on the surface to make multifunctional scaffolds that combine mechanical support with therapeutic delivery.^10^

Among electrospinnable polymers, PCL remains a preferred polymer in tissue engineering for its favourable mechanical strength, biocompatibility, and gradual degradation profile.^1,11^ PCL degrades slowly, which ensures mechanical stability of scaffolds over prolonged periods before being replaced with cell secreted neo-matrix. PCL favorable rheological and thermal properties support facile electrospinning and blending with other polymers or bioactive particles.^12^ Although inherently hydrophobic and biologically inert, PCL's physical characteristics are well-suited for soft tissue applications, and its limitations can be mitigated through incorporation of functional additives such as AgNPs.

AgNPs exert potent antimicrobial action across Gram-negative and Gram-positive organisms, including drug-resistant strains. AgNPs bactericidal mechanisms are multi-faceted: reactive oxygen species generation, disruption of cell membranes, enzyme inhibition, and DNA damage, all contributing to effective bacterial cell death and reduced potential for resistance development.^13^ Incorporation of AgNPs into PCL fibers through electrospinning ensures uniform nanoparticle dispersion and creates a controlled-release antimicrobial reservoir in the scaffold.^14,15^ Localized Ag ion delivery directly at the wound interface maintains high antimicrobial local concentration with limited systemic exposure to avoid cytotoxic effects such as argyria or delayed healing.^16^ Optimizing AgNP loading and fiber morphology enables balancing antimicrobial efficacy with biocompatibility, which is critical to the safety and performance of the wound dressing.

A substantial body of research supports the effectiveness of PCL-AgNPs nanofibers.^2,8,17^ For example, Pazos-Ortiz *et al.* (2017) reported that the antibacterial effectiveness of PCL-AgNPs composites increased with AgNP concentration, showing inhibitory effects against *E. coli*, *S. aureus*, *K. pneumoniae*, and *P. aeruginosa*.^18^ Moreover, studies have shown that direct contact between PCL-based mats and bacteria resulted in high antimicrobial action. Other methods, such as sputter-deposition of Ag onto PCL fibers, have also yielded scaffolds with strong antibacterial efficacy. Nonetheless, research gaps remain.^19,20^ Comparative evaluation of embedding methods (*e.g.*, blending *vs.* surface deposition), long-term cytocompatibility, and performance against biofilms or *in vivo* infection models are less frequently addressed. Consequently, there is a pressing need for optimized PCL-AgNPs systems with well-characterized structure–function relationships to ensure high antibacterial efficacy without compromising host tissue safety. The present work develops electrospun PCL nanofibers containing biosynthesized AgNPs at varying concentrations. It examines how nanoparticle incorporation influences fiber morphology, mechanical properties, surface topography, wettability, and antibacterial activity. By combining structural functionality with localized antimicrobial effect, the study introduces a nanofibrous scaffold designed to limit microbial growth at the wound interface.

This study offers remarkable differences compared with other reported PCL/AgNP nanofiber-based systems. This study follows a fully green synthesis route by utilizing Piper nigrum leaf extract – instead of using a chemical reducing agent. This study takes a broadly, but controlled loading window (0.04, 0.4, and 1 wt%) and systematically correlates the AgNP content with the surface roughness, hydrophilicity and the mechanical reinforcement of the mats. This study offers complete morphological, mechanical and surface characterization of the study system under identical conditions in order to examine the structure–function relationships that are inherent in the antibacterial activity against Gram-positive and Gram-negative bacteria. By avoiding inaccurate claims of Ag ion release and *in vivo* healing, this study provides a clean, evidence-based account of how biosynthesized AgNPs modulate the PCL fibers to develop mechanically robust scaffolds for use in wound-dressing applications.

## 2. Materials and methods

### 2.1 Materials

PCL with a MW of 80 000 g mol^−1^ was sourced from a local supplier in Baghdad. Acetone (analytical grade) and silver nitrate (AgNO_3_, ≥99% purity) were acquired for solution preparation and nanoparticle synthesis. Fresh leaves of *Piper nigrum* were obtained from a traditional herbal vendor located in the Baghdad markets and were used as the plant-based reducing agent for green synthesis of AgNPs.

### 2.2 AgNPs synthesis

Approximately 12 g of *Piper nigrum* leaves were thoroughly rinsed, air-dried at ambient temperature, and finely grounded. The resulting powder was transferred to a sterile glass beaker containing 100 mL of deionized water, stirred thoroughly, and passed through Whatman no. 1 filter paper to remove plant residues. The filtrate underwent centrifugation at 10 000 rpm for 10 minutes to enhance clarity. To initiate silver nanoparticle synthesis, 6 mL of the clarified extract was gradually introduced into 75 mL of an aqueous 1 mM AgNO_3_ solution. The mixture was maintained at 70 °C under continuous magnetic stirring for 90 minutes, followed by a brief high-speed agitation lasting 2 minutes. A visible colour shift to pale reddish-brown signified nanoparticle formation, consistent with previous findings.^21^ The reaction mixture was then left to cool naturally, followed by centrifugation at 10 000 rpm for 15 minutes. The isolated AgNPs were washed multiple times with ethanol to eliminate unbound phytochemicals and residual ions, then collected and dried for subsequent application.

### 2.3 Membrane preparation using AgNPs and PCL

**Fig. 1:**
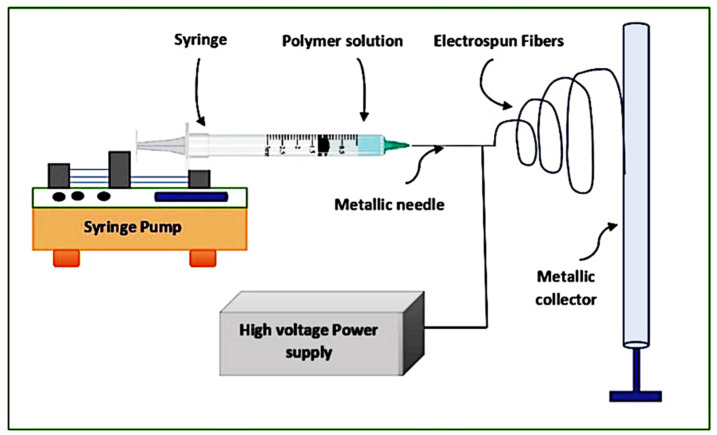
Flowchart illustrating the electrospinning process. Adopted from ref. 20 with no changes.

Biosynthesized AgNPs were incorporated into PCL solutions at 0.04 wt%, 0.4 wt%, and 1 wt%. The appropriate nanoparticle masses were measured using a high-precision analytical balance. Separately, a 13 wt% PCL solution was prepared by dissolving the PCL in chloroform. The dissolution process was carried out in a sealed glass flask under continuous magnetic stirring for five hours to ensure complete homogenization of the polymer. Following the preparation of the polymer-nanoparticle solutions, electrospinning was used to fabricate the nanofibrous membranes. The electrospinning system (Fig. 1) comprised a high-voltage source, syringe pump, and 5 mL syringe fitted with a 12 mm needle. The PCL solution was dispensed at 0.5 mL h^−1^, with a 10 cm gap in-between needle and the grounded collector. Nanofibers were collected on aluminum foil and stored in a desiccator for later analysis.

### 2.4 PCL-AgNPs electrospun scaffold characterization

#### 2.4.1 Scanning electron microscopy (SEM) analysis

SEM was used to verify the presence of hybrid PCL-AgNPs fibres. Samples were cut into 25 mm sections, and a gold coating was applied using a JEOL JFC1600 sputter coater. Imaging was conducted using a JEOL JSM6390 scanning electron microscope operated at 25 kV. Electrospun diameters were calculated *via* ImageJ software.

#### 2.4.2 Atomic force microscopy (AFM)

AFM was used to evaluate the surface topography and roughness of the electrospun PCL-AgNPs nanofibers. Measurements were conducted using an Ambios Universal AFM system (Ambios Technology, CA, USA) operated in contact mode. A silicon nitride (Si–N) cantilever arm was used for all scans under ambient conditions. Acquired topographical images were analyzed using WSxM version 5.0, developed by Nanotec Electronica S. L.^21^ Representative samples from each AgNP concentration group were scanned to determine the influence of nanoparticle loading on fiber surface characteristics. The roughness data were used to assess potential correlations with antimicrobial performance and as surface texture plays a crucial role in antibacterial performance.^22^

#### 2.4.3 Fourier transform infrared spectroscopy (FTIR)

PerkinElmer 2000 spectrometer (Waltham, MA, USA) was used to identify functional groups and assess interactions between PCL and AgNPs. Samples were scanned from 4000 to 500 cm^−1^ at a resolution of 4 cm^−1^. Spectral shifts, intensity changes, and new peaks were examined to confirm nanoparticle incorporation and changes in the PCL matrix.

#### 2.4.4 Mechanical properties measurements

The mechanical properties of the PCL-AgNPs scaffolds were evaluated in accordance with ASTM D882 standards for thin plastic films. Rectangular specimens were prepared with a fixed length of 6 cm and mounted onto the grips of a universal testing machine (Omnitest-25, Mecmesin Corporation, West Sussex, United Kingdom). Testing was performed at a speed of 25 mm min^−1^ until sample fracture. The applied force and elongation were recorded continuously during the test. Stress–strain curves were generated, and key mechanical parameters.

#### 2.4.5 Contact angle measurement

Contact angles were measured at using a goniometer (OCA15EC, DataPhysics, Germany) to assess PCL-AgNPs scaffold wettability. A 5 μL droplet of deionized water was placed on each scaffold, and the angle was measured within 5 seconds. Measurements were taken at three positions per sample to ensure consistency. Values indicated the hydrophilic or hydrophobic nature of the nanofiber surfaces.

#### 2.4.6 Antibacterial effect

*Escherichia coli* (ATCC 12228) and *Staphylococcus aureus* (ATCC 6538-P) were used to assess PCL-AgNPs antibacterial properties. Bacterial cultures were grown in Mueller–Hinton broth (MHB) at 37 °C. After incubation, 100 μL of each bacterial suspension was spread onto the surface of MHB agar plates. Electrospun PCL fibres containing different concentrations of AgNPs were cut into 6 mm discs and placed on the inoculated plates. The plates were then incubated overnight at 37 °C. The antibacterial performance of the scaffolds were evaluated by calculating the inhibition zones.

## 3. Results and discussion

### 3.1 SEM analysis

**Fig. 2:**
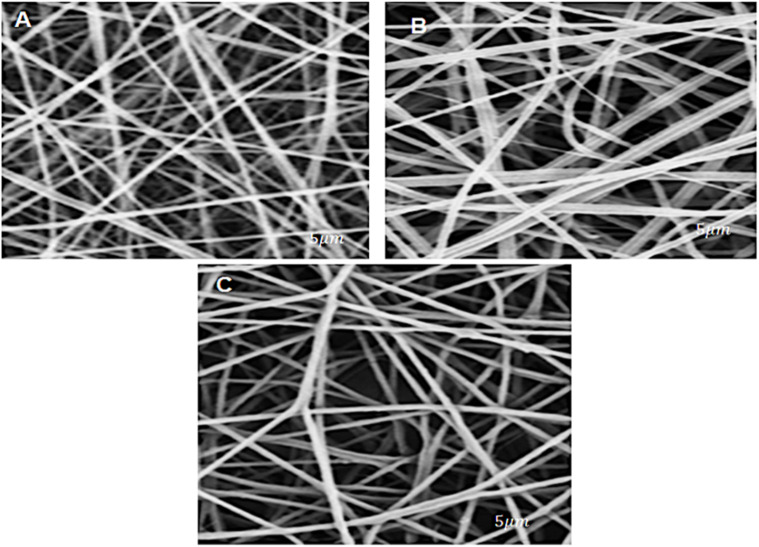
SEM images of (A) neat PCL; (B) PCL-0.4%AgNPs; and (C) PCL-1%AgNPs scaffolds.

SEM was used to evaluate the morphology of electrospun nanofibers (Fig. 2). Image A shows neat PCL nanofibers, while images B and C correspond to PCL-0.04%AgNPs and PCL-1%AgNPs, respectively. All samples exhibited a uniform fiber network without any observable bead formation, indicating stable electrospinning conditions and proper PCL jet elongation.

The addition of AgNPs influenced fiber morphology and led to a consistent reduction in average fiber diameter. Pure PCL fibers displayed an average diameter of 1669 nm. When 0.04 wt% AgNPs were incorporated, the mean diameter decreased to 1459 nm. A further decrease to 830 nm was recorded at 1 wt% AgNP concentration, consistent with earlier findings.^23^ The reduction in fiber diameter with increasing silver content may be attributed to changes in solution conductivity. Higher nanoparticle concentrations tend to increase the electrical charge density of the PCL solution, which enhances the stretching forces during electrospinning and results in finer fiber formation.

All SEM micrographs showed well-aligned and smooth fibers without visible agglomeration of AgNPs on the surface. The absence of surface defects and the homogenous distribution of fibers suggest successful AgNPs incorporation into the PCL matrix. The improved uniformity and finer diameters may contribute positively to the scaffold's surface-to-volume ratio, which is a critical parameter in cellular interactions and antibacterial activity. The morphological features observed support the potential of the PCL-AgNPs scaffolds for biomedical applications that require both structural consistency and surface functionality.

### 3.2 AFM analysis

**Fig. 3:**
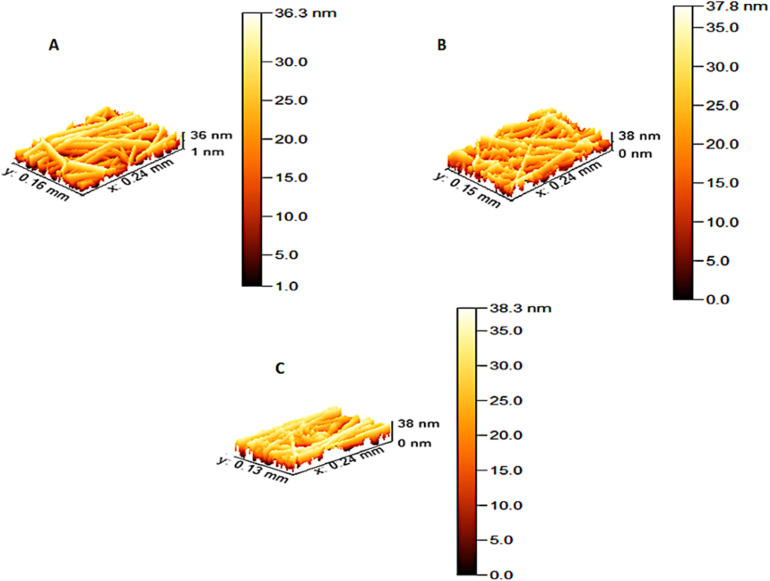
AFM topography of (A) neat PCL; (B) PCL-0.4%AgNPs; and (C) PCL-1%AgNPs scaffolds. Increased AgNPs concentration correlates with elevated surface roughness and greater topographical heterogeneity across the scaffold surface.

The surface topography of PCL nanofibers and PCL-AgNPs scaffolds was evaluated using AFM. Fig. 3 presents the three-dimensional AFM images, while quantitative roughness values are listed in Table 1. All samples exhibited characteristic fibrous surface structures with visible nanoscale topographical variation.

**Table 1:** Surface roughness values (mean ± SD) of PCL-AgNPs scaffolds measured by ATM, ranked from highest to lowest based on AgNPs concentration

Sample	Roughness average Sa (nm)	Root mean square Sq (nm)	Ten points height Sz (nm)	Average diameter (nm)
Neat PCL	7.87197	9.08587	35.3725	47.08236
PCL-0.4%AgNPs	7.38951	8.68156	37.7843	48.92903
PCL-1%AgNPs	8.7398	10.0639	38.2667	51.24387

A gradual increase in surface roughness was observed with increasing AgNPs concentration. The roughness of the neat PCL scaffold measured 2.69 ± 0.31 nm. For the PCL-AgNPs nanocomposites, the RMS roughness values increased to 3.61 ± 0.43 nm and 4.27 ± 0.35 nm for 0.4 wt% and 1 wt% AgNPs, respectively. The increase in roughness may be credited to the presence and distribution of AgNPs within the PCL matrix, which introduces localized surface irregularities. Similar trends were reported by Hassan *et al.* (2017),^24^ who demonstrated a positive correlation between nanoparticle loading and nanofiber surface topography. Surface roughness plays a critical role in modulating biological responses at the cell–material interface. Increased roughness has been shown to enhance scaffold wettability, protein adsorption, and initial cell attachment, increased roughness has been shown to enhance scaffold wettability, protein adsorption, and initial cell attachment, all of which are essential for tissue integration. Zamani *et al.* (2017) observed that nanoscale roughness influences cellular adhesion and proliferation in a manner dependent on both cell type and substrate features.^25^ Martins *et al.* (2019) further noted that specific roughness ranges may support or inhibit pathological cell migration, such as the behavior of bone tumor cells on uneven surfaces.^26^

The topographical features observed in the PCL-AgNPs nanocomposites suggest improved surface functionality for biological applications. Nanostructured roughness may contribute not only to enhanced cell adhesion but also to increased surface energy, which may disrupt microbial colonization and improve antibacterial effectiveness. These findings support the multifunctionality of the PCL-AgNPs nanofibrous scaffolds in biomedical applications where both tissue integration and infection control are essential.

### 3.3 FT-IR analysis

**Fig. 4:**
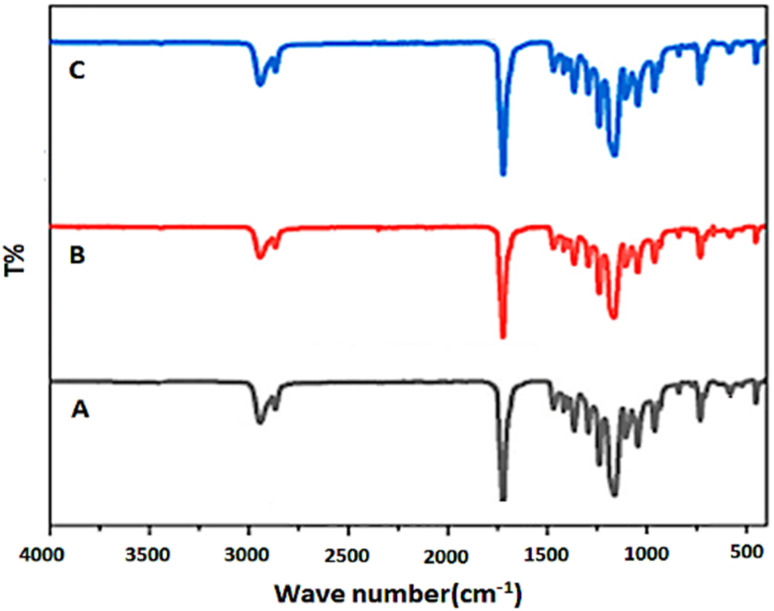
FT-IR spectra of (A) neat PCL; (B) PCL-0.4%AgNPs; (C) PCL-1%AgNPs scaffolds.

FT-IR spectroscopy was performed to investigate chemical modifications and identify functional groups present within the electrospun PCL and PCL-AgNPs nanofiber scaffolds. Fig. 4 presents the FT-IR spectra for neat PCL (A), PCL-0.4%AgNPs (B), and PCL-1%AgNPs (C). All spectra revealed characteristic absorption bands of PCL, including strong peaks near 1720 cm^−1^ attributed to the stretching vibration of carbonyl (C=O) groups and peaks near 1160–1290 cm^−1^ corresponding to C–O and C–C stretching vibrations within the PCL backbone. The intensity and position of these peaks remained consistent across all samples, which confirm that AgNPs addition did not alter the chemical identity of the PCL matrix.

The introduction of AgNPs led to the appearance of additional broad absorption bands between 3200 and 3700 cm^−1^. These bands can be attributed to O–H stretching vibrations, likely originating from hydroxyl-containing groups present in the green-synthesized nanoparticles or absorbed moisture. A prominent band near 1633 cm^−1^ was also observed, indicating the presence of H–O–H bending modes associated with water molecules. Similar findings suggested presence of surface-bound hydroxyl groups were linked to enhanced hydrophilicity and antimicrobial activity.^23,27^

Although PCL is inherently biocompatible, its hydrophobic nature may limit certain biomedical applications. The appearance of O–H and H–O–H related bands suggests that AgNPs incorporation introduces hydrophilic features into the polymeric scaffold. Improved hydration may enhance surface interactions with aqueous media and support antibacterial effectiveness. In parallel, increased surface polarity and available hydroxyl groups can disrupt bacterial membranes and reduce microbial adhesion. All together contribute to the scaffold's antibacterial performance.

### 3.4 Tensile strain analysis

**Fig. 5:**
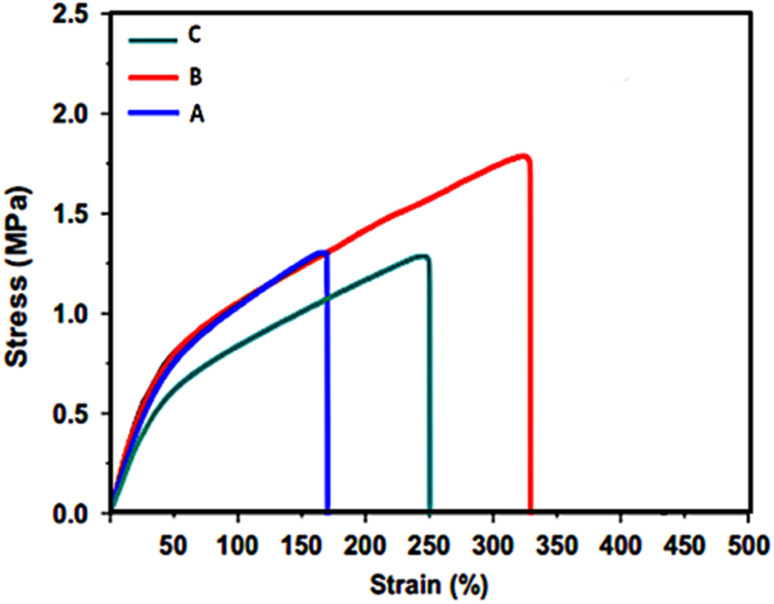
Tensile stress–strain curves of (A) neat PCL; (B) PCL-0.4%AgNPs; and (C) PCL-1%AgNPs scaffolds. Curves illustrate variations in stiffness and elongation as a function of nanoparticle concentration, with increased modulus at moderate Ag content and decreased mechanical performance at higher loading.

The mechanical properties of the electrospun PCL and PCL-AgNPs nanofiber scaffolds were evaluated under uniaxial tensile loading. All samples demonstrated an initial elastic region followed by progressive deformation until fiber failure (Fig. 5). The incorporation of AgNPs affected both the tensile strength and elongation of the nanofibrous scaffolds.

Neat PCL fibers (curve A) exhibited moderate tensile strength and high elongation at break, consistent with the inherent ductility of the PCL. Upon incorporating 0.4 wt% AgNPs (curve B), a notable improvement in the tensile modulus was observed, indicating enhanced stiffness. The tensile performance increase may be attributed to favorable interactions between the PCL matrix and the uniformly distributed nanoparticles. These interactions contribute to stress transfer efficiency and mechanical reinforcement within the scaffold. At 1 wt% AgNP loading (curve C), a decline in tensile strength and elongation was recorded. The reduction in mechanical integrity may result from nanoparticle agglomeration at higher concentrations, which creates localized stress concentration zones within the fiber structure. According to Valerini *et al.* (2020), nanoparticle clusters can act as defect sites that disrupt the continuity of stress transfer from the polymer to the fillers.^28^ Such behavior explains the decreased tensile performance observed at higher Ag content. Poor dispersion also compromises the structural uniformity of the nanofibers and limits their ability to bear mechanical loads. The results suggest that moderate incorporation of AgNPs (0.4 wt%) enhances mechanical properties, while excessive loading induces structural weaknesses. These findings emphasize the need for optimal nanoparticle concentration to balance mechanical reinforcement with other functional properties such as antibacterial activity.

### 3.5 Contact angle measurement

**Fig. 6:**
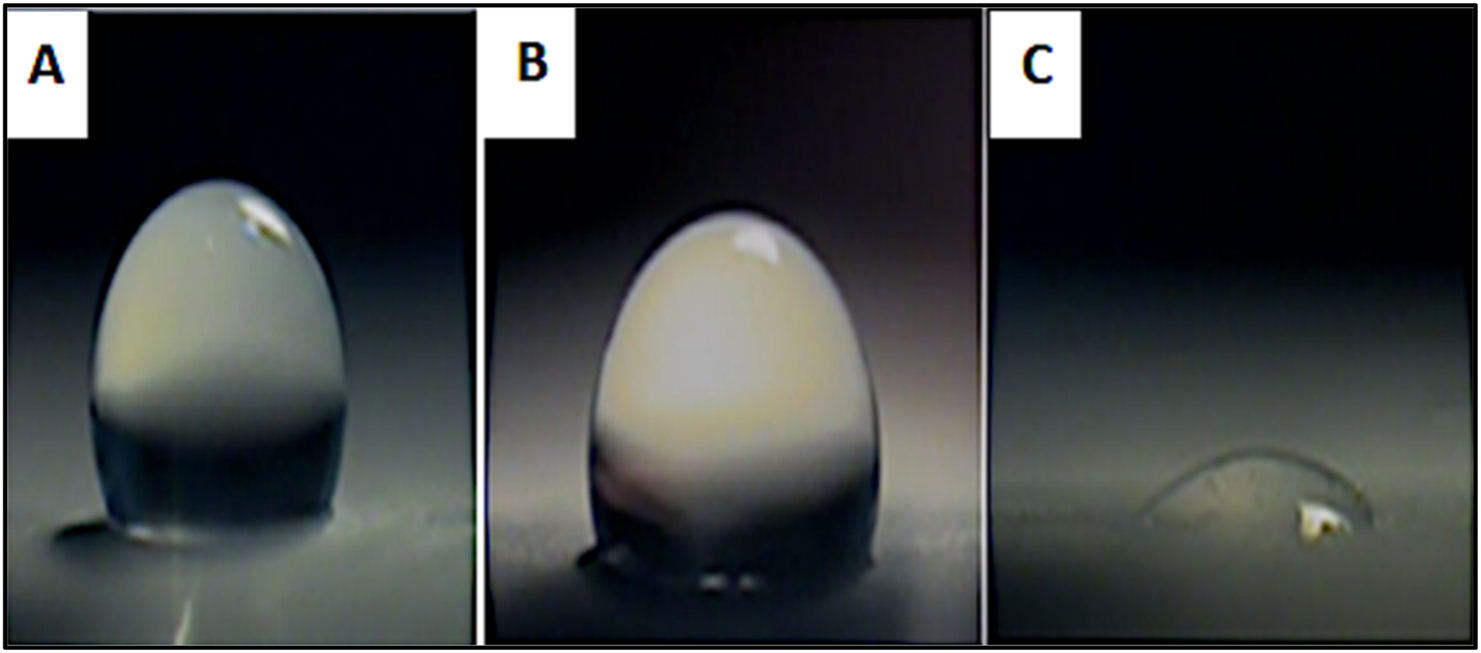
Static water contact angle images of electrospun nanofiber scaffolds: (A) neat PCL, (B) PCL-0.4%AgNPs, and (C) PCL-1%AgNPs.

Contact angle measurements were performed to evaluate the surface wettability of neat PCL nanofibers and PCL-AgNPs composite scaffolds. The results reveal the influence of AgNPs incorporation on the hydrophilic or hydrophobic nature of the electrospun materials (Fig. 6 and Table 2).

**Table 2:** Contact angles of electrospun scaffolds (mean ± SD, *n* = 3)

Samples	Contact angles (°)
PCL	106.4 ± 4.3°
PCL-0.4%AgNPs	95.4 ± 5.3°
PCL-1%AgNPs	66.5 ± 4.2°

The neat PCL nanofiber mat exhibited a WCA of 106.4 ± 4.3°, indicating a highly hydrophobic surface. Such behavior is attributed to the presence of non-polar methylene (–CH_2_–) groups in the PCL molecular structure, which contribute to low surface energy. The contact angle remained stable over time, with no significant decrease observed after 10, 60, or 120 seconds, confirming the limited interaction between water droplets and the pure PCL surface.

With the addition of 0.4 wt% AgNPs, the WCA decreased to 95.4 ± 5.3°, suggesting a partial improvement in surface wettability. At 1 wt% AgNPs loading, the WCA was further reduced to 66.5 ± 4.2°, demonstrating a clear transition from hydrophobic to hydrophilic behavior. The change may be attributed to the polar nature of silver oxide and the introduction of surface-bound oxygen-containing groups during nanoparticle synthesis and fiber formation. These modifications likely increase the surface energy and promote water adsorption.

Earlier studies support these observations, reporting similar hydrophilic shifts in PCL nanofibers upon AgNPs incorporation.^15,18,29^ The enhanced wettability is expected to improve cellular adhesion and may also contribute to antibacterial efficacy by facilitating aqueous phase interactions with microbial membranes interactions with microbial membranes.

### 3.6 Antibacterial activity

**Fig. 7:**
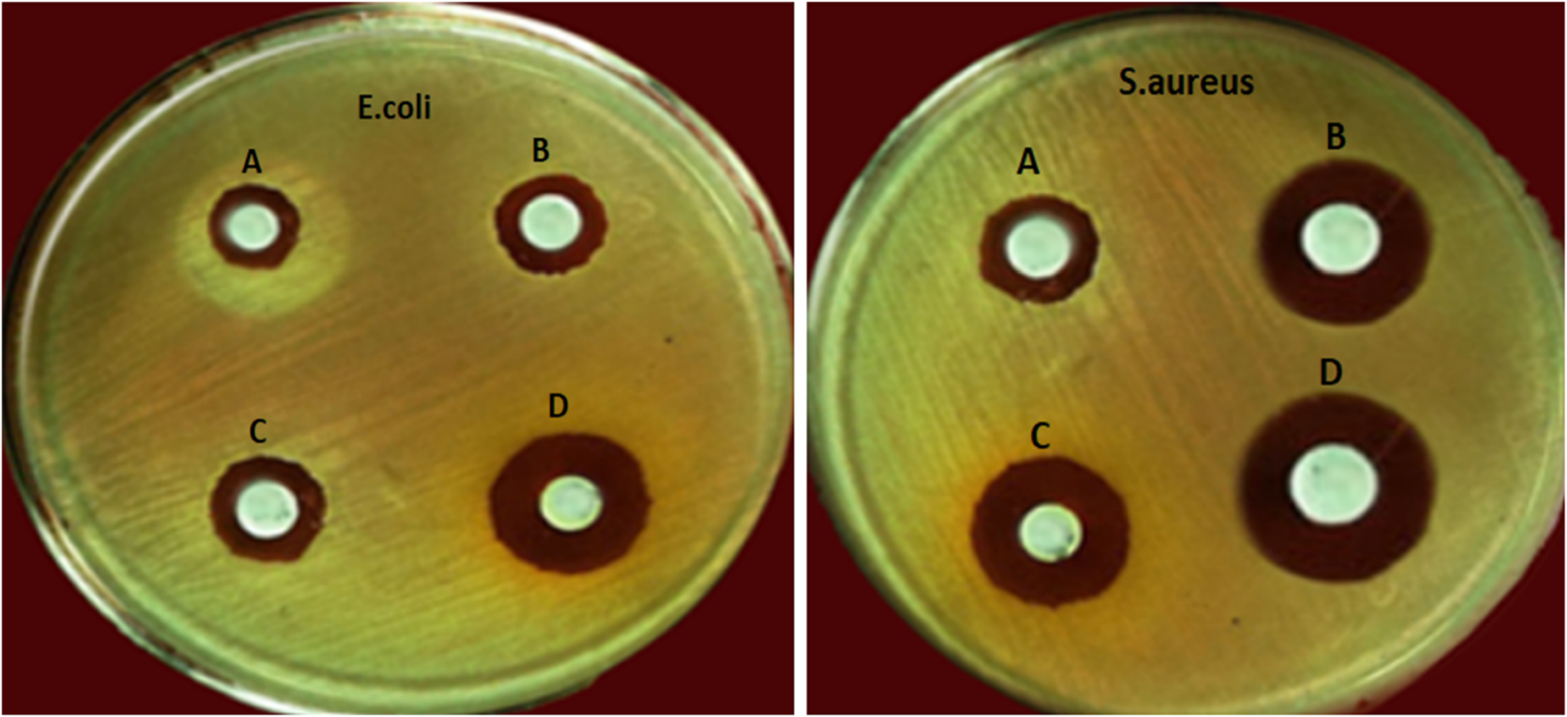
Agar diffusion assay showing antibacterial inhibition zones around electrospun scaffolds: (A) neat PCL; (B) PCL-0.04%AgNPs; (C) PCL-0.4%AgNPs; (D) PCL-1%AgNPs.

The antibacterial activity of PCL-AgNPs scaffolds was evaluated *via* agar disc diffusion method against *E. coli* (Gram-negative) and *S. aureus* (Gram-positive). Inhibition zones are shown in Fig. 7, with corresponding measurements summarized in Table 3.

**Table 3:** Zones of inhibition (mean ± SD, mm) for *E. coli* and *S. aureus* exposed to PCL nanofiber membranes loaded with AgNPs at varying concentrations

Sample	Inhibitory zone (mm)	
	*E.coli*	*S.aureus*
PCL-0.04%AgNPs	8.2 ± 0.4	10.4 ± 0.3
PCL-0.4%AgNPs	8.5 ± 0.7	9.5 ± 0.6
PCL-1%AgNPs	17.5 ± 0.6	18.6 ± 0.5

Neat PCL fibers (Group A) exhibited no inhibition zones, confirming their lack of intrinsic antimicrobial properties. In contrast, PCL scaffolds loaded with AgNPs showed clear zones of inhibition against both tested strains, indicating effective antimicrobial activity. At 0.04 wt% AgNPs (Group C), the inhibition zones measured 8.2 ± 0.4 mm for *E. coli* and 10.4 ± 0.3 mm for *S. aureus* (*P* = 0.0380 and *P* = 0.0063, respectively), reflecting early-stage activity at low nanoparticle concentration. A modest increase in antimicrobial effect was observed at 0.4 wt% AgNPs (Group B), with inhibition zones of 8.5 ± 0.7 mm against *E. coli* and 9.5 ± 0.6 mm against *S. aureus* (*P* = 0.0068 and *P* = 0.0059, respectively). The most pronounced antibacterial effect occurred at 1 wt% AgNPs (Group D), where inhibition zones expanded significantly to 17.5 ± 0.6 mm and 18.6 ± 0.5 mm for *E. coli* and *S. aureus*, respectively (*P* = 0.0072 and *P* = 0.0044). These results demonstrate a concentration-dependent antibacterial response.

The enhanced inhibition against *S. aureus* is likely due to structural differences in bacterial cell walls. The thick peptidoglycan layer in Gram-positive bacteria allows more direct interaction with silver ions, contributing to greater susceptibility. In contrast, Gram-negative bacteria, such as *E. coli*, have an additional outer membrane enriched with lipopolysaccharides, which can act as a barrier and delay ion penetration.^8,30,31^ The results confirm that silver-loaded PCL nanofibers exhibit potent and tunable antibacterial properties. These findings support the use of such scaffolds in biomedical applications where infection prevention is essential.

## 4. Conclusion

Electrospun nanofibrous membranes composed of PCL and biosynthesized AgNPs were successfully fabricated and characterized for antibacterial applications. Increasing AgNP content enhanced surface roughness and hydrophilicity while influencing fiber morphology and mechanical strength. Optimal mechanical reinforcement was achieved at 0.4 wt% AgNPs; however, further addition led to nanoparticle aggregation and weakened structural integrity. FTIR analysis confirmed the chemical incorporation of AgNPs and indicated improved water affinity. Contact angle analysis supported the transition from hydrophobic to hydrophilic behavior, which enhances antibacterial performance. The antibacterial results showed concentration-dependent inhibition against *E. coli* and *S. aureus*, with the strongest activity observed at 1 wt% AgNPs. These findings confirm that AgNP-incorporated PCL nanofibers hold strong potential for use in antibacterial biomaterial development and infection control.

## Author contributions

Authors contributed equally.

## Conflicts of interest

The authors declare no conflicts of interest.

## Data Availability

The datasets generated and analyzed during this study are available from the corresponding author on reasonable request.
